# Organic geochemical analysis of archaeological medicine pots from Northern Ghana. The multi-functionality of pottery

**DOI:** 10.1016/j.jas.2012.03.015

**Published:** 2012-07

**Authors:** Sharon E. Fraser, Timothy Insoll, Anu Thompson, Bart E. van Dongen

**Affiliations:** a School of Earth, Atmospheric and Environmental Sciences, University of Manchester, Manchester M13 9PL, UK; b Williamson Research Centre for Molecular Environmental Science, University of Manchester, Manchester M13 9PL, UK; c School of Arts, Histories and Cultures, University of Manchester, Manchester, M13 9PL, UK; d School of Environmental Sciences, The University of Liverpool, Liverpool, L69 3GP, UK

**Keywords:** GC–MS, GC–C–IRMS, Compound specific isotopic analysis, Medicine pots, C_3/_C_4_ plants, Ghana

## Abstract

Sherds from pots found layered under a granite boulder in the Tong Hills of the Upper East Region of Northern Ghana seem, based on their deposition context to have been used for the preparation of medicines. Organic geochemical and isotopic analyses of these sherds and a modern day analogue reveal an *n*-alkanoic acid composition that is consistent with their being used in the preparation of plant derived substances. Isotopic analyses of the modern medicine pot indicate a contribution of *n*-alkanoic acids derived from plants that use C_4_ carbon fixation, most likely maize, sorghum and/or millet suggesting that this pot was used for cooking C_4_ based plant substances, perhaps, based on current analogy, staple porridge type food. The modern medicine pot could thus have had a prior use. The absence of C_4_ plant residues in the archaeological sherds suggests that either staple foodstuffs differed radically to today, or, more likely, were not prepared in vessels that were to be used for medicinal purposes.

## Highlights

► First organic geochemical and isotopic analyses of Ghana medicine pots. ► Analyses indicating a usage in the preparation of plant derived substances. ► Analyses of modern analogue indicates usage in cooking C_4_ based plant substances. ► Medicine was a more firmly bounded and demarcated domain of practice than today.

## 1 Introduction

**Fig. 1:**
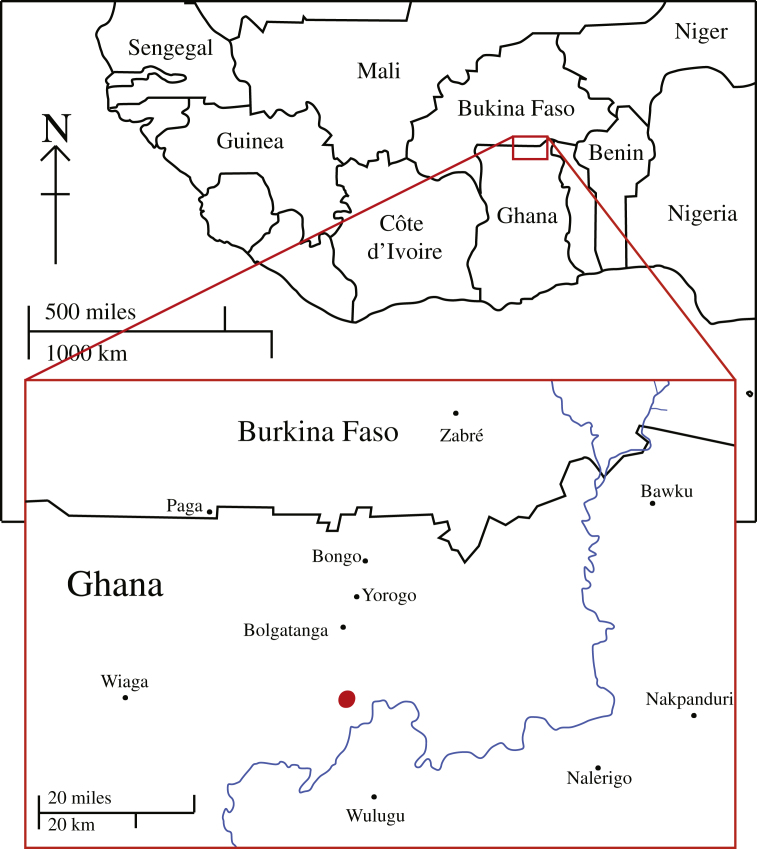
Map showing the Upper East Region of Ghana, indicating the location of the Tong Hills.

Pottery plays an important role in the preparation of medicines by the Talensi of the Tong Hills of the Upper East Region in Northern Ghana (Fig. 1). Pots are used both to prepare and store medicines (Insoll, 2011b). In 2008 a context was identified during an archaeological survey in Touwang in Tamboog section that was seemingly linked with the disposal of pots used for the preparation of medicines (Insoll, 2011b). This was subsequently excavated (Insoll et al., 2008). The unit from which the archaeological medicine pots were recovered was one of twenty excavations of varying sizes that have been completed in different contexts; seven shrine units, one unit in the medicinal pot disposal feature, six in abandoned compounds and settlement areas, three in rock shelters and rock features, one in an abandoned blacksmiths forge, and two in an iron smelting site (Insoll, 2010; Insoll et al., 2008). The 13 OSL dates obtained from the Research Laboratory for Archaeology and the History of Art at Oxford University range between 2500 ± 235 (726–256 BC) for a unit near the Yaane shrine (TONN 08 B 1) and 435 ± 50 (AD 1523–1623) for a unit in a collapsed earthen house (TOU 08 (A) 6) (see Insoll et al., in press for full details). An OSL date was obtained from the archaeological medicine pot feature from a sample recovered from a depth of 20 cm (TOU 08 (C) 2) and this was of 500 ± 45 (AD 1463–1553; RLAHA Sample X3336).

The Talensi now occupy the Tong Hills but oral tradition suggests that their ethnogenesis is unlikely to predate the mid-seventeenth century at the earliest (Fortes, 1945, 1949; Insoll et al., in press). Hence, although the archaeological medicine pots cannot be directly linked with the Talensi ethno-linguistic group for the formation of their ethnic identity post-dates the context, a comprehensive programme of ethnographic research in the Tong Hills was undertaken to gain an idea of medicinal substance preparation and use (Insoll, 2011b). This modern medicine data collected for analogical (Insoll, 2011b), and analytical purposes (van Dongen et al., 2011) indicated that a range of substances are used as medicines in the area today and include minerals (e.g. clays), plants, and animal parts and products (Insoll, 2011b). Previously no information existed on medicine use amongst the Talensi and as far as the authors are aware (Insoll, 2011a), neither medicine pots nor associated equipment from either archaeological or other contexts in sub-Saharan Africa have previously been the focus of organic geochemical or isotopic analyses.

*Clay-based medicines* – These formed part of the group of 10 modern medicines identified that were prepared either wholly or predominantly from substances other than plants (see Table 3 Insoll, 2011b). Clay medicines are prepared by simply mixing clays that have been collected from shrine sites with water (Insoll, 2011b). Heat is not normally used as an adjunct to this process, which is best described as geophagic consumption, or cold-water dissolution and external application. Previous analysis of these clays has shown that they are naturally occurring and that they have not been altered by the addition of other materials (van Dongen et al., 2011).

*Plant based medicines* – A total of 33 modern plant based medicines have been identified. (see Table 2 Insoll, 2011b for information about individual species and their medicinal use). These can be made from a single plant or by mixing up to 5 different plants. They utilise many parts of the plants e.g. seeds, juice, stem, leaves, bark, roots, which are ground into a powder and then charred or boiled, before either drinking or applying externally to affected areas of the body (Insoll, 2011b).

*Animal based medicines* – These types of medicine are also still in use (see Table 3 Insoll, 2011b). They are prepared by charring and grinding animal bones, usually rabbit and bird bones, and the resulting powder is then inhaled or put in incisions (Insoll, 2011b).

As heating is normally applied in both the preparation of plant medicines and food, it is believed that lipids will have been absorbed into the fabric of the pottery. As shown by other studies of archaeological pottery, it is possible to obtain analytical data related to pottery use from a combination of organic geochemical and compound specific isotopic analysis (CSIA; Berstan et al., 2008; Charters et al., 1993; Copley et al., 2005a; Evershed et al., 2002, 2008; Giorgi et al., 2010; Gregg and Slater, 2010; Romanus et al., 2007). The ratio of C_16:0_ to C_18:0_ *n*-alkanoic acids, for instance, is often used as an indicator of either a plant or animal origin for fats which have been extracted from pottery e.g. Copley et al. (2005b) and Evershed et al. (2002). If the C_16:0_ *n*-alkanoic acid (palmitic) is more abundant than the C_18:0_ *n*-alkanoic acid (stearic) then it is suggested that the sample is of plant fat origin (Copley et al., 2005b), whereas if the C_18:0_ *n*-alkanoic acid (stearic) is more abundant then the acids have an animal origin. This ratio may not be constant over archaeological time, indicating that the amount of C_16:0_ *n*-alkanoic acid could increase relative to the C_18:0_ homologue as it can be produced by the break down of the C_18:1_ *n*-alkanoic acid (Mills and White, 2006: 34). In addition, palmitic acid can be preferentially leached from pottery into the surrounding soil as it is more water soluble than stearic acid (Steele et al., 2010). Therefore CSIA, in particular the δ^13^C_16:0_ *n*-alkanoic acid vs. δ^13^C_18:0_ *n*-alkanoic acid compositions, is becoming more commonly used to distinguish between the origins of animal derived *n*-alkanoic acids (e.g. Berstan et al., 2008; Copley et al., 2005b; Gregg and Slater, 2010; Romanus et al., 2007) as well as between animal and plant derived material (Steele et al., 2010).

The aims of this project were therefore, using a combination of organic and compound specific isotope analysis (CSIA) of the archaeological medicine pot samples and the modern medicine pot as a comparison, to determine if *n*-alkanoic acids have been absorbed into the fabric of the pottery during its use. If this is the case then the results of the analyses can be used to determine the plant and/or animal origin of the acids and to assess whether or not these pots were used for the preparation of, for example, food prior to their use in the preparation of medicines, to assess whether their single function attribution is in fact valid.

## 2 Materials and methods

### 2.1 Samples

**Fig. 2:**
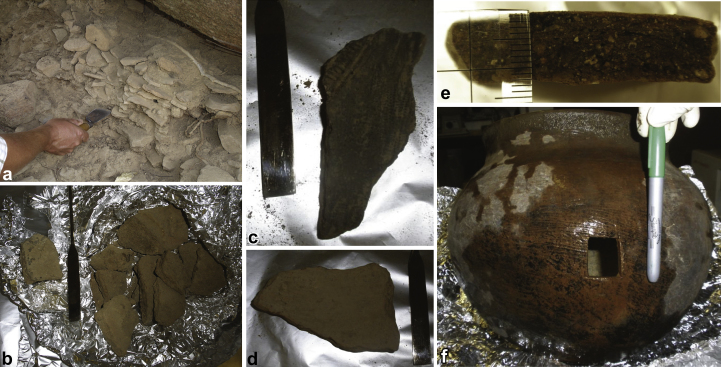
The medicine pot sherds recovered from Touwang (TOU 08 (C)). a) TOU 08 (C). The sherds in situ underneath the large Bongo granite boulder; b) pot sherds from context TOU 08 (C) 7 prior to cleaning; c) a single sherd from TOU 08 (C) 7 after cleaning; d) a sherd from TOU 08 (C) 4 after cleaning; e) cross section of a sherd from TOU 08 (C) 5; f) modern sample from Tamboog.

Four pot sherds from the Touwang archaeological site and a modern medicine pot, a *yanba-dok*, purchased from the *Tengdana*, the Earth Priest of Tamboog, were chosen for organic analysis. Based on the context in which the pot sherds were found at the Touwang site, multiply layered and hidden in a cleft under a large Bongo granite boulder (Insoll et al., 2008 and Insoll, 2011b), it is likely, according to local informants, that the pots were used in the preparation of medicines, and were hidden away because they were perceived as dangerous and/or polluting (Fig. 2a). The context was described of as a mechanism for getting rid of vessels that you did not necessarily want people to see (K. Tendaan pers. comm. 19/3/08). The hard surface deposits that sloped down from the boulder were cleaned and the uppermost sherds delimited (TOU 08 (C) 1). This was extended further down the slope of deposits (TOU 08 (C) 2). In total, the pot deposits were approximately 30 cm deep.

The archaeological samples used in this study were chosen based on their visual differences e.g. surface decoration, thickness and colour (Table 1) and they therefore represent different pots that may also have been used for different medicines. The modern comparison was provided by the complete pot from Tamboog that was known to have been used in the preparation of medicines. This pot is called a *yanba-dok* and is used in conjunction with a *laa* or lid that can also be used for medicine grinding (see below). The *yanba-dok* is a flared mouth bowl with a simple out-turned rounded rim and with a rim diameter of 19.3 cm. It is decorated with a pale reddish orange slip on the exterior and partially on the interior. The exterior also has crude incised decoration of multiple overlapping lines.

The sherds for analysis were removed with a trowel and wrapped immediately in aluminium foil and then stored in a sealed metal canister for transport to the UK. Subsequently, before organic geochemical analysis the surfaces of the archaeological pot sherd samples were cleaned in the laboratory with a small hand drill (Dremel 300 with a sanding tip) to remove any soil which may have adhered during burial and/or contamination from modern human contact (Fig. 2c and d).

### 2.2 Extraction method

The cleaned samples were ground up, extracted, hydrolysed and derivatized, comparable to methods described elsewhere (Evershed et al., 1999; Copley et al., 2005a; Mukherjee et al., 2008). In short, 5 g of each sample was ultrasonically extracted using a mixture of chloroform and methanol (2:1, v/v). The obtained total lipid extracts were filtered through a silica column to remove any particles and 20 μl of internal standard (deuterated tetracosane) was added to an aliquot before hydrolysis with methanolic sodium hydroxide (5%) – heated for 1 h at 70 °C. The extract was cooled and acidified to pH 3 with hydrochloric acid (1 M). Prior to analyses the extracts were derivatized with BF_3_ in MeOH (70 °C for 1 h) to convert acids into their corresponding methyl esters. The resulting dried extracts, after the removal of excess reagents under a gentle stream of nitrogen, were dissolved in hexane and analysed using Gas Chromatography–Mass Spectrometry (GC–MS) and Gas Chromatography–Combustion–Isotope Ratio Mass Spectrometry (GC–C–IRMS). Blanks were also analysed with the samples to ensure that no contamination was introduced during the preparation procedure and were found to be less than 1:100 for all target analytes.

The residues remaining after extraction were further saponified to allow any remaining compounds to be extracted from the pottery sherd. The method used was a combination of that used by Craig et al. (2004) and Giorgi et al. (2010). 3.5 g of the extracted residue was placed in a screw top vial with 6 ml of methanolic sodium hydroxide (5%), heated for 4 h at 70 °C, and analysed using the same protocols as the extracted lipids.

### 2.3 Instrumental analysis

GC–MS was performed using a Agilent 7890A GC, equipped with an Agilent 7683B autosampler and programmable temperature vaporization (PTV) inlet, interfaced to an Agilent 5975C MSD mass spectrometer operated in electron ionization (EI) mode (scanning a range of *m/z* 50–600 at 2.7 scans s^−1^, ionisation energy 70 eV and a solvent delay of 3 min). The heated interface temperature and PTV inlet were set at 280 °C with the EI source temperature at 230 °C and the MS quadruple at 150 °C. Analyses were performed using an HP-5 MS capillary column (J&W Scientific; 5% diphenyl-dimethylpolysiloxane; 30 m, 250 μm id, 0.25 μm film thickness). The samples were run at constant flow (1 ml/min) with helium as the carrier gas. The oven temperature was programmed from 70 °C to 130 °C at 20 °C min^−1^, then to 300 °C at 4 °C min^−1^, with a hold time of 25 min.

GC–C–IRMS was performed at the University of Liverpool on a Delta V Advantage (Thermo Fisher, Bremen) mass spectrometer linked to a Trace Ultra GC with a ConFlo IV interface. Samples in hexane/ethyl acetate were loaded on a TriPlus autosampler and 1 μl injected in splitless mode on a DB5 fused silica column (30 m, 250 μm id, 0.25 μm film thickness, J&W Scientific). The effluent from the GC passed immediately into and through a combustion reactor consisting of a NiO tube with CuO/NiO wires which was held at 1030 °C. The effluent then passed through a water separator consisting of a Nafion tube prior to entering the mass spectrometer. The GC programme was ramped from 45 °C (1 min) to 290 °C at 4 °C min^−1^ (20 min hold time). The injector was held at 300 °C. Ultra high purity grade helium was used as the carrier gas at a constant flow of 1.4 ml/min. Isodat 3 software was used to automatically compute the ^13^C/^12^C and ^18^O/^16^O ratios of each sample peak, referenced to the standard CO_2_ gas and its known ^13^C/^12^C and ^18^O/^16^O content. Carbon isotopic compositions represent averaged values of duplicate or triplicate analyses. The CO_2_ reference gas was externally calibrated relative to Vienna Pee Dee Belemnite (VPDB) on SIRA.

## 3 Results

### 3.1 GC–MS

**Fig. 3:**
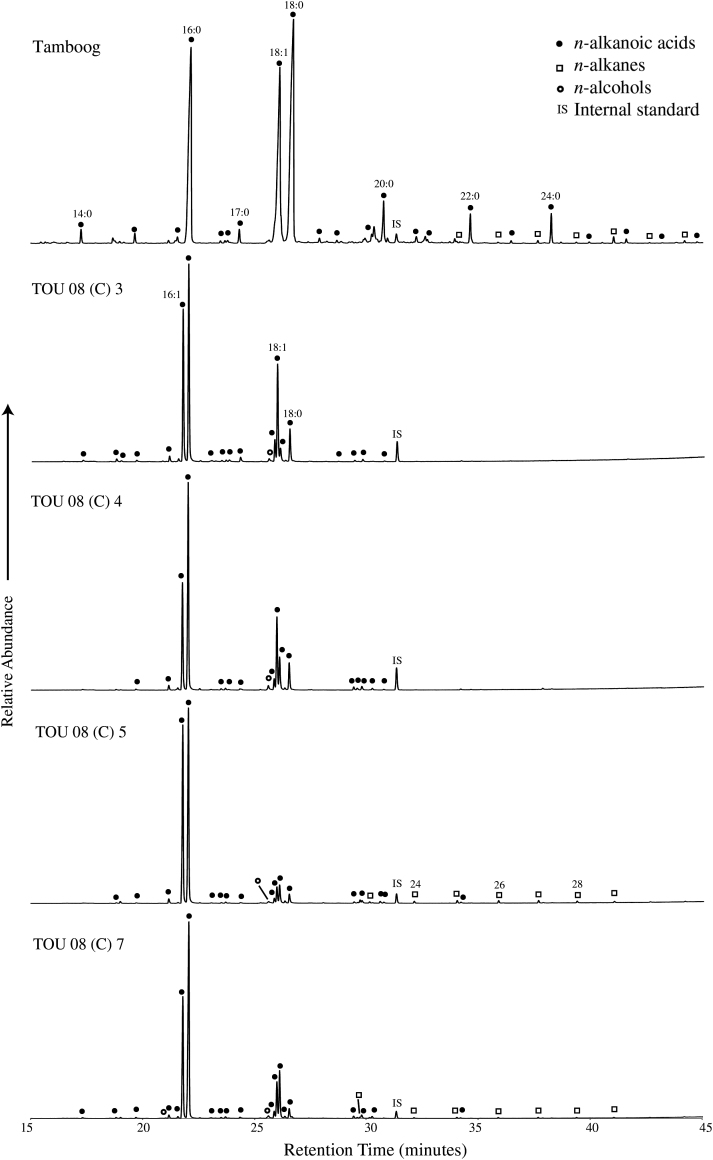
A comparison of the relative abundance of compound extracts from the modern medicine pot sample (Tamboog) and archaeological samples TOU 08 (C) 3, 4, 5 and 7 (The tallest peak in each chromatogram is equal to 100% and not an absolute concentration). The numbers above the peaks refer to the length of carbon chain: the number of double bonds present. 16:0 and 18:0 *n*-alkanoic acids are palmitic and stearic acid, respectively.

The extracts of all archaeological sherds were dominated by a series of short to mid-chain (C_12_–C_20_) *n*-alkanoic acids, with the C_16:0_ homologue being the most abundant (Table 2 and Fig. 3). Other dominant components were the C_16:1_, C_18:1_ and C_18:0_ homologues. The total amount of short to mid-chain *n*-alkanoic acids (C_14_–C_20_) ranged from 8.3 to 27 μg/g sample. In addition, small concentrations of long chain *n*-alkanes were found in TOU 08 (C) 5 and 7 (Fig. 3). Compared to the archaeological sherds the total amount of short chain *n*-alkanoic acids in the modern pot sherd was substantially higher, 58 μg/g sherd (Table 2). The distribution pattern was also different, with the C_18:0_ being the most abundant component, followed by the C_16:0_ and the C_18:1_ (Table 2 and Fig. 3). Besides these mid-chain length *n-*alkanoic acids, substantial amounts of long chain *n*-alkanoic acids, up to C_28_, were also present, with a clear even over odd chain length predominance. The re-extracted residues of the pot sherds were also dominated by *n*-alkanoic acids with distribution patterns comparable to that of the original analysis e.g. dominated by C_16:0_, C_18:0_ and C_18:1_ *n*-alkanoic acids, with smaller amounts of *n*-alcohols (not shown), but overall concentrations were much lower (up to 60 times). This indicates that the original extraction was sufficient to liberate the majority of the compounds of interest and that, as suggested by Evershed (2008) these cannot be distinguished from contamination as the concentrations are below 5 μg/g sherd.

### 3.2 Compound specific isotope analysis

Compound specific isotopic analyses indicated that of the five samples analysed, only TOU 08 (C) 3, TOU 08 (C) 4 and the modern sample from Tamboog had sufficient concentrations of C_16:0_ and C_18:0_ to give reliable δ^13^C values (Table 2). In the case of samples TOU 08 (C) 5 and 7, the concentrations of C_18:0_ were too low to give reliable δ^13^C values. The δ^13^C_18:0_ values of the two remaining archaeological sherds and the modern Tamboog pot sample were comparable with average values ranging from −26.8‰ to −25.7‰. The δ^13^C_16:0_ values were comparable between all archaeological samples, with average values ranging from −28.8‰ to −27.0‰; however the modern sample was more enriched in δ^13^C_16:0_ with a value of −21.3‰.

## 4 Discussion

### 4.1 Source/origin of *n*-alkanoic acids

Information on the lipid markers preserved in the Ghana medicine pots is limited. Our results illustrate that such lipids, (*n*-alkanoic acids), can be used to evaluate the source of organic material prepared in these pots. Although there are some differences among the sherds analysed, all 4 archaeological sherds generally have comparable *n*-alkanoic acid distribution patterns dominated by the C_14_ to C_20_ homologues. A lack of the very short carbon chains (e.g. less than C_14_) is not unexpected, as these can be preferentially removed by dissolution and evaporation. It is further possible that some of these fats were degraded during burial making it difficult to distinguish between some types of fats as their degraded chemical profile looks very similar (Dudd and Evershed, 1998).

The dominance of palmitic acid compared to stearic acid suggests that a significant proportion of the alkanoic acids present is of plant origin. Indeed, these distribution patterns are comparable to those observed in other pottery dominated by plant derived material, such as pottery lamps from Qasr Ibrim, Egypt (Copley et al., 2005b), a late Bronze age sherd of red lustrous wheelmade ware from Saqqara, Egypt (Steele et al., 2010) and a pot produced from Revere clay and limestone used to cook maize kernels and coarse corn meal in a laboratorial based experimental setup (Reber and Evershed, 2004b). However, an animal contribution/origin cannot be completely excluded as it is possible that due to degradation during burial C_18:1_ can break down into C_16:0_ causing a higher than original C_16:0_ acid (Mills and White, 2006: 34). In addition Reber and Evershed (2004b) showed that maize lipids absorbed in pot sherds decompose quickly and therefore after a reasonably short period of time the acid profile becomes indistinguishable from other plant and/or meat origins. Another factor that must be taken into consideration is that the *n*-alkanoic acids preserved in the archaeological sherds could also be affected by leaching after burial, causing a reduction in the amount of *n*-alkanoic acids preserved. The area where the archaeological samples were collected from is known for periodically being subjected to heavy rainfall, particularly between July and September, when it is the height of the rainy season and the majority of the mean annual precipitation of 100–115 cm takes place. This is when leaching potentially could have occurred and the archaeological deposits situated under the granite boulder might have been affected. Interestingly, past research indicated that C_16:0_ *n*-alkanoic acids are preferentially removed affecting the relative distribution patterns (e.g. Early Neolithic pot sherds from Somerset - Berstan et al., 2008). However, preferential removal of the C_16:0_ *n*-alkanoic acid would suggest that the original composition was even more dominated by this acid, making it highly unlikely that this process has significantly affected the distribution patterns.

To further clarify the origin of the *n*-alkanoic acids present the δ^13^C values of these acids were determined and compared with previous data for specific sources such as equine, ruminant (goat, sheep and cow/buffalo) and chicken adipose *n*-alkanoic acids (Copley et al., 2003; Dudd and Evershed, 1998 and Dudd et al., 1999) and C_3_ plant oils (Steele et al., 2010, Fig. 4). Several sources, such as fish (Craig et al., 2007), diary and pig products (Dudd and Evershed, 1998) were less likely to be present and have therefore not been used in this comparison. Marine fish were excluded since the location of the study site is relatively far away from any marine environment making it unlikely that marine fish would have been used in the area. However fresh-water fish from the White Volta River could not be completely excluded as a potential source (Fortes and Fortes, 1936). The presence of dairy products is also unlikely because their consumption is very rare, with, for instance, curdled milk described as a “luxury food” by Fortes and Fortes (1936: 260). Some cattle are present in the Tong Hills but no instances of the consumption of dairy products were seen during the seven years of fieldwork in the Tong Hills (2004–2011), and it is unlikely that dairy products consumption was any more frequent in the past. Pigs are also a relatively recent introduction to the Tong Hills (H. Goldaan pers. comm. 4/11/08) and are precluded from Talensi sacrifice on account of this, as animals unknown to the ancestors (Insoll, 2010: 234), and as a consequence are unlikely to have been consumed in the Tong Hills at the time the pottery recovered from Touwang was in use. A general absence of faunal remains because of sacrificial distribution precludes examining this source of evidence (Insoll, 2010).

The δ^13^C values of the C_16:0_ and C_18:0_ *n*-alkanoic acids of the archaeological samples TOU 08 (C) 3 and 4 both plot in the area suggested by Steele et al. (2010) for plant *n*-alkanoic acids and as such supports the results of the organic geochemical analysis, indicating that these sherds indeed contain predominantly plant derived material. Considering that the other two archaeological samples, TOU 08 (C) 5 and 7 have comparable distribution patterns and the δ^13^C values of the C_16:0_ and C_18:0_ *n*-alkanoic acids are similar (Table 2) it is most likely that the origin of these acids is the same as the other two archaeological samples, e.g. plant derived.

**Fig. 4:**
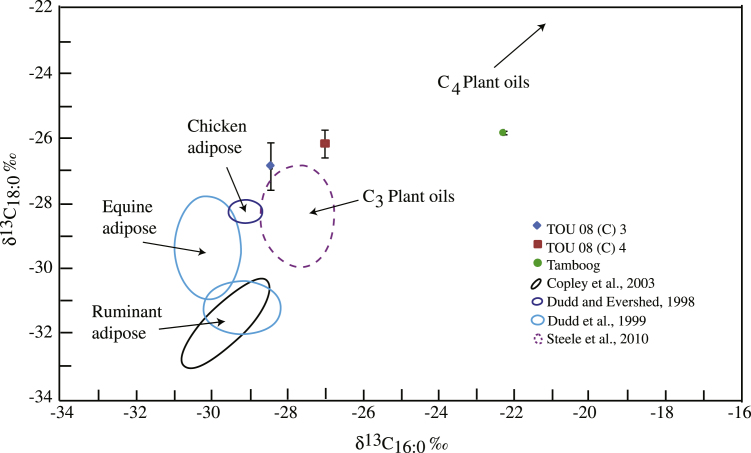
Carbon isotopic ratios of C_16:0_ and C_18:0_*n*-alkanoic acids in lipid extracts of modern material and archaeological pot sherds shown with previous data from Copley et al. (2003), Dudd and Evershed (1998), Dudd et al. (1999), and Steele et al. (2010).

Compared to the archaeological samples the *n*-alkanoic acid distribution pattern of the modern day analogue from Tamboog showed a different distribution pattern with the C_18:0_ *n*-alkanoic acid being the dominant acid present (Fig. 3). Previously, a C_18:0_ over C_16:0_ *n*-alkanoic acid dominance has been attributed to an animal origin of the *n*-alkanoic acids (Copley et al., 2005b and Evershed et al., 2002). This suggests a different, animal dominated, origin for the *n*-alkanoic acids present in the modern pot if compared to the archaeological samples. However, the isotopic composition of the C_16:0_ and C_18:0_ *n*-alkanoic acids do not support this origin. Particularly the C_16:0_ acid is substantially more enriched in δ^13^C if compared to those generally observed in fats of animal origin (Copley et al., 2003; Dudd and Evershed, 1998; Dudd et al., 1999) or C_3_ plants (Steele et al., 2010, Fig. 4). The most logical explanation is that, unlike the archaeological samples (C_3_ plants), this pot was used for the preparation of C_4_ plant based substances. C_4_ plants have a different mechanism for carbon fixation compared to C_3_ plants and are generally more enriched in δ^13^C (Bender, 1968; Reber and Evershed, 2004a; Reber et al., 2004; Sage and Monson, 1999 and van der Merwe and Tschauner, 1999). The analysis of modern maize (a C_4_ plant) by Reber and Evershed (2004a) for instance indicates δ^13^C values for both C_16_ and C_18_ *n*-alkanoic acids between −17 and −16‰ and between −17 and −12.5‰, respectively (shown in Fig. 4 of this paper and Fig. 5 in Reber and Evershed, 2004a). The isotopic signature of the modern pot is intermediate between that of the C_3_ plants from the archaeological pot sherds and that of maize in Reber and Evershed (2004a), suggesting a mixed contribution of C_3_ and C_4_ plants. C_3_ and C_4_ plants cannot be distinguished based solely on the *n*-alkanoic acids distribution patterns, particularly if preservation/degradation effects are taken into consideration (Reber et al., 2004). It has been suggested that a mixture of different fatty acids from different sources within one pot can produce a signature of a completely different fat which was actually never present (Reber and Evershed, 2004a), which could be the case with the modern pot. The mixture of C_3_ and C_4_ plants might have created a false positive for animal fats when looking at only the *n*-alkanoic acids distribution patterns.

To summarise, organic geochemical analyses and compound specific isotope analyses indicate that the *n*-alkanoic acids in both the archaeological sherds and the modern day analogue are most likely of plant origin, with a substantial C_4_ plant contribution to the modern pot.

### 4.2 Pottery usage prior to utilisation as medicine pots?

The results of the organic analyses indicate that the archaeological pots have been used for the preparation of plant based medicines and/or plant based food, but not of the staple porridge type found today and made from millet, sorghum or maize (cf. Casey, 2000: 34; Fortes and Fortes, 1936). Based on the *n*-alkanoic distribution pattern it is not possible to distinguish between these specific uses, but based on the carbon isotopic compositions it is highly unlikely that animal based food has been prepared in these pots. The contemporary Talensi medicine data indicates that prepared plant based medicine is added to a millet based meal in two instances, whereas millet as a medicine ingredient was only found in relation to one non-plant based medicine where it was placed whole and unmodified inside a calabash along with other substances. Millet also formed part of the sacrifices that either precede or follow healing in relation to seven examples of plant based medicines (Insoll, 2011b, Tables 2 and 3), but these form part of the chain of accompanying ritual actions rather than the pot related medicine preparation processes. *Sorghum bicolor* was also used as an ingredient in one plant based medicine where it was ground along with a legume (indet.), and soil from an anthill into a paste to be used as a poultice or ointment (Insoll, 2011b, Table 2). This is the sole C_4_ plant use that can potentially be linked with a pot for the *laa*, the pot lid used in conjunction with the larger *yanba-dok* can be utilised for medicine grinding. Whether this is also used for the larger quantities of coarser substances (e.g. anthill soil) required for medicines such as this poultice is not known, though it can be assumed as unlikely as the life of the *laa* would be shortened.

Hence, it is possible that the modern medicine pot was either used for the preparation of C_4_ based foodstuffs of the staple porridge type and thus had a prior life to its use as a medicine pot, or it was used for the preparation of meals to which medicines were added, and thus has always been a medicine pot. Either way, it differs in its usage pattern from the archaeological examples. Even if it was used for the preparation of a medicine involving a C_4_ plant it still differs significantly from the four archaeological medicine pot samples that do not show such a usage, suggesting differences in medicine practices and substances over time.

There are approximately 8000 different species of C_4_ plant (Sage and Monson, 1999) but it is thought that they can be traced back to 6 main linkages in warm, semi arid and arid areas of Earth (Sage et al., 2011). Of these millet is thought to have been first domesticated in the Sahara and sorghum in northeast Africa (Sudan-Chad area). Casey (2000: 22) notes that the earliest evidence for sorghum in West Africa is a single seed dated to approximately 4000 bp from Adrar Bous in Niger. Domesticated millet has been recorded at both Kursakata in northern Nigeria and Oursi in Burkina Faso dated to c. 3000 bp (Casey, 2000: 22), whereas maize was first domesticated in Mexico (van der Merwe and Tschauner, 1999). Maize was not introduced to Africa until the 16th Century by Portuguese traders, and became a common crop in Western Africa by the 1700's (Casey, 2000; van der Merwe and Tschauner, 1999). The absence of C_4_ plant residues in the Touwang sherds indicates that they were not used for preparing plant based foodstuffs of maize/millet/sorghum types prior to their deposition, unlike the seemingly multi-functional modern medicine pot, hence the absence of a C_4_ plant contribution in these sherds.

The C_4_ plants: maize (*Zea mays*), sorghum (*Sorghum bicolor*) and several species of millet make up 70% of the cereals grown in Africa (FAO, 1994). Maize is commonly used in modern day Ghana to make “koko” which is fermented maize dough made in to a porridge or “kenkey” where the dough is made into a ball, wrapped in leaves and boiled for about 3 h (Annan et al., 2003a,b. See Halm et al., 1996 for description of cooking methods). In Northern Ghana millet is the frequently preferred C_4_ plant and along with sorghum is ground and used to prepare *tuo zafi* or TZ, the starchy porridge staple (Casey, 2000: 34).

## 5 Conclusions

Organic geochemical and isotopic analyses of archaeological sherds from medicine pots recovered from Touwang in the Tong Hills reveal an *n*-alkanoic acid composition that is consistent with their being used in the preparation of plant derived substances. No evidence for the use of animal fats is observed. Analyses of the modern medicine pot analogue indicate a contribution of *n*-alkanoic acids derived from plants that use C_4_ carbon fixation, most likely maize, sorghum, and/or millet. C_4_ plants form part of the healing processes, but their linkages with medicine pots are not primary. This suggests that the modern medicine pot might have had a prior use. This use pattern differs considerably from the archaeological examples that lack C_4_ residues suggesting that staple foodstuffs either differed radically, or, more likely, were not prepared in vessels that would then be used for medicinal purposes. Excluding maize, the common usage of which post-dates the archaeological pot context, other domesticated C_4_ plants were available but these were not prepared in the archaeological medicine pots. This suggests a conscious choice was made to separate food from medicine, and also indicates the significant differences between the ethnographic and archaeological data.

Potentially, this also indicates, as the specific hidden deposition context also suggests, that medicine was a more firmly bounded and demarcated domain of practice than it is today, or alternatively that it has changed over time and in so doing has incorporated the staple foodstuffs as an agent for medicine ingestion. In summary, the potential for further organic geochemical and isotopic analyses of medicine substances and equipment from archaeological contexts in sub-Saharan Africa is significant for it could allow the diachronic dimension to be more fully explored.

## Figures and Tables

**Table 1:** Descriptions of archaeological and modern day medicine pottery samples from the Tong Hills region, Ghana.

	TOU 08 (C) 3	TOU 08 (C) 4	TOU 08 (C) 5	TOU 08 (C) 7	Tamboog
External Colour	Traces of dark brown slip over orange fabric	Orange brown fabric that possibly originally had a dark brown slip	Band of black slip, otherwise mid-brown fabric colour	Dark brown/Black slip	Reddish orange slip, smoke? Stained black in places
Decorations	Indeterminate roulette	Fine cord wrapped stick roulette	Cord wrapped stick roulette	Two parallel incised lines	Incised overlapping lines across the surface
Thickness of sherd	15 mm (max)	10 mm	12 mm	8 mm	5 mm
Internal Colour	Traces of dark brown slip	As fabric colour but traces of possible dark brown slip	Lighter orange slip on interior	Dark brown/black slip	Dark brown to black
Internal fabric	Poorly sorted cream angular grains up to 2 mm in size in a fine orange clay matrix	A few larger angular grains up to 1 mm in a fine orange brown clay matrix	Very poorly sorted coarse mid-brown fabric with several different types of angular grains up to 2 mm in size	Very poorly sorted angular to sub angular grains up to 3 mm in size (low sphericity) in a clay matrix	Larger sub angular cream grains with a low sphericity and up to 1 mm in size in a mid to dark brown clay matrix. Also some darker patches which could be either mineral grains or burnt organic matter.
					
Hardness	<2.5 (finger nail)	<2.5	<2.5	<2.5	<3.5 (copper coin)

**Table 2:** Concentrations of compounds extracted from the studied pottery samples and carbon isotopic compositions for selected compounds.

	TOU 08 (C) 3	TOU 08 (C) 4	TOU 08 (C) 5	TOU 08 (C) 7	Tamboog
*Concentrations* (μg/g *sediment*)					
C_16:0_	4.0^a^	3.6^a^	8.4^a^	13^a^	15^a^
C_16:1_	4.8	1.8^a^	7.2	6.9^a^	0.41^a^
C_18:0_	0.63	0.47	0.40	0.49	20
C_18:1_	2.2^a^	1.9^a^	1.5^a^	4.8^a^	15
C_18:2_	0.40	0.18	0.19	0.39	0.19
∑ short chain *n*-alkanoic acids^b^	11	8.4	19	27	58
∑ long chain *n*-alkanoic acids^c^	bdl^d^	bdl^d^	0.06	0.17	4.1
∑ *n*-alkanes	bdl^d^	bdl^d^	0.56	0.30	0.27
∑ *n*-alcohols	bdl^d^	0.10	0.09	0.21	0.26
*Carbon isotopic composition (‰)*					
δ^13^C_16:0_	−28.75 ± 0.03	−26.99 ± 0.02	−28.12 ± 0.02	−28.51 ± 0.05	−21.26 ± 0.02
δ^13^C_18:0_	−26.79 ± 0.74	−26.06 ± 0.41	bdl^d^	bdl^d^	−25.70 ± 0.05

a Summed concentration of isomers.

b Summed concentration of C_14_–C_20_*n*-alkanoic acids.

c Summed concentration of C_21_–C_28_*n*-alkanoic acids.

d bdl – below detection limit.

## References

[bib1] Annan N.T., Poll L., Plahar W.A., Jakodsen M. (2003). Aroma characteristics of spontaneously fermented Ghanaian maize dough for *kenkey*. Eur. Food. Res. Technol..

[bib2] Annan N.T., Poll L., Sefa-Dedeh S., Plahar W.A., Jakobsen M. (2003). Volatile compounds produced by *Lactobacillus fermentum, Saccharomyces cerevisiae* and *Canadida krusei* in single starter culture fermentation of Ghanaian maize dough. J. Appl. Microbiol..

[bib3] Bender M. (1968). Mass spectrometric studies of carbon 13 variations in corn and other grasses. Radiocarbon.

[bib4] Berstan R., Stott A.W., Minnitt S., Bronk Ramsey C., Hedges R.E.M., Evershed R.P. (2008). Direct dating of pottery from its organic residues: new precision using compound-specific carbon isotopes. Antiquity.

[bib5] Casey J. (2000). The Kintampo Complex. BAR S906.

[bib6] Charters S., Evershed R.P., Goad L.J., Leyden A., Blinkhorn P.W., Denham V. (1993). Quantification and distribution of lipid in archaeological ceramics: implications for sampling potsherds for organic residue analysis and classification of vessel use. Archaeometry.

[bib7] Copley M.S., Berstan R., Dudd S.N., Docherty G., Mukherjee A.J., Straker V., Payne S., Evershed R.P. (2003). Direct chemical evidence for widespread dairying in prehistoric Britain. PNAS.

[bib8] Copley M.S., Berstan R., Dudd S.N., Staker V., Payne S., Evershed R.P. (2005). Dairying in antiquity. I. Evidence from absorbed lipid residues dating to the British Iron Age. J. Archaeol. Sci..

[bib9] Copley M.S., Bland H.A., Rose P., Horton M., Evershed R.P. (2005). Gas chromatographic, mass spectrometric and stable carbon isotope investigations of organic residues of plant oils and animal fats employed as illuminants in archaeological lamps from Egypt. Analyst.

[bib10] Craig O.E., Love G.D., Isaksson S., Taylor G., Snape C.E. (2004). Stable carbon isotopic characterisation of free and bound lipid constituents of archaeological ceramic vessels released by solvent extraction, alkaline hydrolysis and catalytic hydropyrolysis. J. Anal. Appl. Pyrolysis..

[bib11] Craig O.E., Foster M., Andersen S.H., Koch E., Crombé P., Milner N.J., Stern B., Bailey G.N., Heron C.P. (2007). Molecular and isotopic demonstration of the processing of aquatic products in northern European prehistoric pottery. Archaeometry.

[bib12] Dudd S.N., Evershed R.P. (1998). Direct demonstration of milk as an element of archaeological economies. Science.

[bib13] Dudd S.N., Evershed R.P., Gibson A.M. (1999). Evidence for varying patterns of exploitation of animal products in different prehistoric pottery traditions based on lipids preserved in surface and absorbed residues. J. Archaeol. Sci..

[bib14] Evershed R.P. (2008). Experimental approaches to the interpretation of absorbed organic residues in archaeological ceramics. World. Archaeol..

[bib15] Evershed R.P., Dudd S.N., Charters S., Mottram H., Stott A.W., Raven A., van Bergen P.F., Bland H.A. (1999). Lipids as carriers of anthropogenic signals from prehistory. Philos. Trans. R. Soc. Lond. B. Biol. Sci..

[bib16] Evershed R.P., Dudd S.N., Copley M.S., Berstan R., Stott A.W., Mottram H., Buckley S.A., Crossman Z. (2002). Chemistry of archaeological animal fats. Acc. Chem. Res..

[bib17] Evershed R.P., Copley M.S., Dickson L., Hansel F.A. (2008). Experimental evidence for the processing of marine animal products and other commodities containing polyunsaturated *n*-alkanoic acids in pottery vessels. Archaeometry.

[bib18] FAO (1994).

[bib19] Fortes M. (1945). (1969). The Dynamics of Clanship Among the Tallensi.

[bib20] Fortes M. (1949). (1967). The Web of Kingship Among the Tallensi.

[bib21] Fortes M., Fortes S. (1936).

[bib22] Giorgi G., Salvini L., Pecci A. (2010). The meals in a Tuscan building yard during the Middle Age. Characterization of organic residues in ceramic potsherds. J. Archaeol. Sci..

[bib23] Gregg M.W., Slater G.F. (2010). A new method for extraction, isolation and transesterification of free n-alkanoic acids from archaeological pottery. Archaeometry.

[bib24] Halm M., Osei-Yaw A., Hayford A.E., Kpodo K.A., Amoa-Awua W.K.A. (1996). Experiences with the use of starter culture in the fermentation of maize for ‘kenkey’ production in Ghana. World. J. Microbiol. Biotech..

[bib25] Insoll T. (2010). Talensi animal sacrifice and its archaeological implication. World. Archaeol..

[bib26] Insoll T. (2011). Introduction. Shrines, substances and medicine in sub-Saharan Africa: archaeological, anthropological, and historical perspectives. Anthropol. Med..

[bib27] Insoll T. (2011). Substance and materiality? The archaeology of talensi medicine shrines and medicinal practices. Anthropol. Med..

[bib28] Insoll T., MacLean R., Kangpeyeng B. (March–April 2008). Excavations and surveys in the Tongo Hills, Upper East region, and Birifor, Upper West region, Ghana. Nyame Akuma.

[bib29] Insoll, T., MacLean, R., Kankpeyeng, B., in press. Temporalising Anthropology: Archaeology in the Talensi Tong Hills. Africa Magna Verlag, Frankfurt.

[bib30] Mills J.S., White R. (2006). The Organic Chemistry of Museum Objects.

[bib31] Mukherjee A.J., Gibson A.M., Evershed R.P. (2008). Trends in pig product processing at British Neolithic Grooved Ware sites traced through organic residues in potsherds. J. Archaeol. Sci..

[bib32] Reber E.A., Evershed R.P. (2004). How did the Mississippians prepare maize? The application of compound-specific carbon isotope analysis to absorbed pottery residues from several Mississippi Valley sites. Archaeometry.

[bib33] Reber E.A., Evershed R.P. (2004). Identification of maize in absorbed organic residues: a cautionary tale. J. Archaeol. Sci..

[bib34] Reber E.A., Dudd S.N., van der Merwe N.J., Evershed R.P. (2004). Direct detection of maize in pottery residues via compound specific stable carbon isotope analysis. Antiquity.

[bib35] Romanus K., Poblome J., Verbeke K., Luypaerts A., Jacobs P., De Vos D., Waelkens M. (2007). An evaluation of analytical and interpretative methodologies for the extraction and identification of lipids associated with pottery sherds from the site of Sagalassos, Turkey. Archaeometry.

[bib36] Sage R.F., Monson R.K. (1999). C_4_ Plant Biology.

[bib37] Sage R.F., Christin P.-A., Edwards E.J. (2011). The C_4_ plant linkages of planet Earth. J. Exp. Bot..

[bib38] Steele V.J., Stern B., Stott A.W. (2010). Olive oil or lard?: distinguishing plant oils from animal fats in the archaeological record of the eastern Mediterranean using gas chromatography/combustion/isotope ratio mass spectrometry. Rapid. Commun. Mass. Spectrom..

[bib39] van der Merwe N.J., Tschauner H., Sage R.F., Monson R.K. (1999). C_4_ plants and the development of human societies. C_4_ Plant Biology.

[bib40] van Dongen B., Fraser S.E., Insoll T. (2011). The composition and origin of Ghana medicine clays. Anthropol. Med..

